# Practical Compass of Single-Cell RNA-Seq Analysis

**DOI:** 10.1007/s11914-023-00840-4

**Published:** 2023-11-29

**Authors:** Hiroyuki Okada, Ung-il Chung, Hironori Hojo

**Affiliations:** 1 Center for Disease Biology and Integrative Medicine, Graduate School of Medicine, The University of Tokyo, Bunkyo-Ku, Tokyo, 113-8655 Japan; 2 Department of Orthopaedic Surgery, The University of Tokyo, Tokyo, Japan; 3 Department of Oral Medicine, Infection, and Immunity, Harvard School of Dental Medicine, Boston, MA 02115 USA; 4 Department of Bioengineering, Graduate School of Engineering, The University of Tokyo, Tokyo, Japan

**Keywords:** Single cell RNA-seq, Transcriptome, Dry analysis, Computational analysis, Practical compass

## Abstract

**Purpose of Review:**

This review paper provides step-by-step instructions on the fundamental process, from handling fastq datasets to illustrating plots and drawing trajectories.

**Recent Findings:**

The number of studies using single-cell RNA-seq (scRNA-seq) is increasing. scRNA-seq revealed the heterogeneity or diversity of the cellular populations. scRNA-seq also provides insight into the interactions between different cell types. User-friendly scRNA-seq packages for ligand-receptor interactions and trajectory analyses are available. In skeletal biology, osteoclast differentiation, fracture healing, ectopic ossification, human bone development, and the bone marrow niche have been examined using scRNA-seq. scRNA-seq data analysis tools are still being developed, even at the fundamental step of dataset integration. However, updating the latest information is difficult for many researchers. Investigators and reviewers must share their knowledge of in silico scRNA-seq for better biological interpretation.

**Summary:**

This review article aims to provide a useful guide for complex analytical processes in single-cell RNA-seq data analysis.

## Introduction

The number of research articles using single-cell RNA-seq (scRNA-seq) is increasing. scRNA-seq has become a core technique in biology in the last 10 years [1]. scRNA-seq enabled us to determine the quantity of each type of mRNA at a single-cell resolution. There are two major reasons why the use of scRNA-seq has spread worldwide. First, scRNA-seq clarifies the heterogeneity or diversity of cell populations from the perspective of gene expression patterns. Second, scRNA-seq can predict the interactions and connectivity between cells, which cannot be easily specified in traditional ways.

scRNA-seq has been used in skeletal biology [2, 3]. For example, we can determine the cell differentiation stages of osteoclasts [4] and their interspecies differences [5•]. Ligand receptor analysis can predict drug repositioning candidates for fracture healing [6] and clarify the hidden mechanisms of ectopic ossification [7]. Gene regulatory analysis has also revealed epigenetic properties in a model of human bone development [8•] and the bone marrow niche [9]. Furthermore, a new subcellular sequencing tool to identify therapeutic targets has been proposed in the field of skeletal biology [10•].

Currently, in silico analysis is necessary not only for computational biologists but also for wet-lab biologists and well-established reviewers [11, 12]. In this review, we have summarized *the *in silico scRNA-seq framework. Active learners can understand the standard workflow and pitfalls of in silico analysis. In addition, this review may be useful to busy reviewers. This article provides a list of points for reviewing of scRNA-seq studies.

**Fig. 1:**
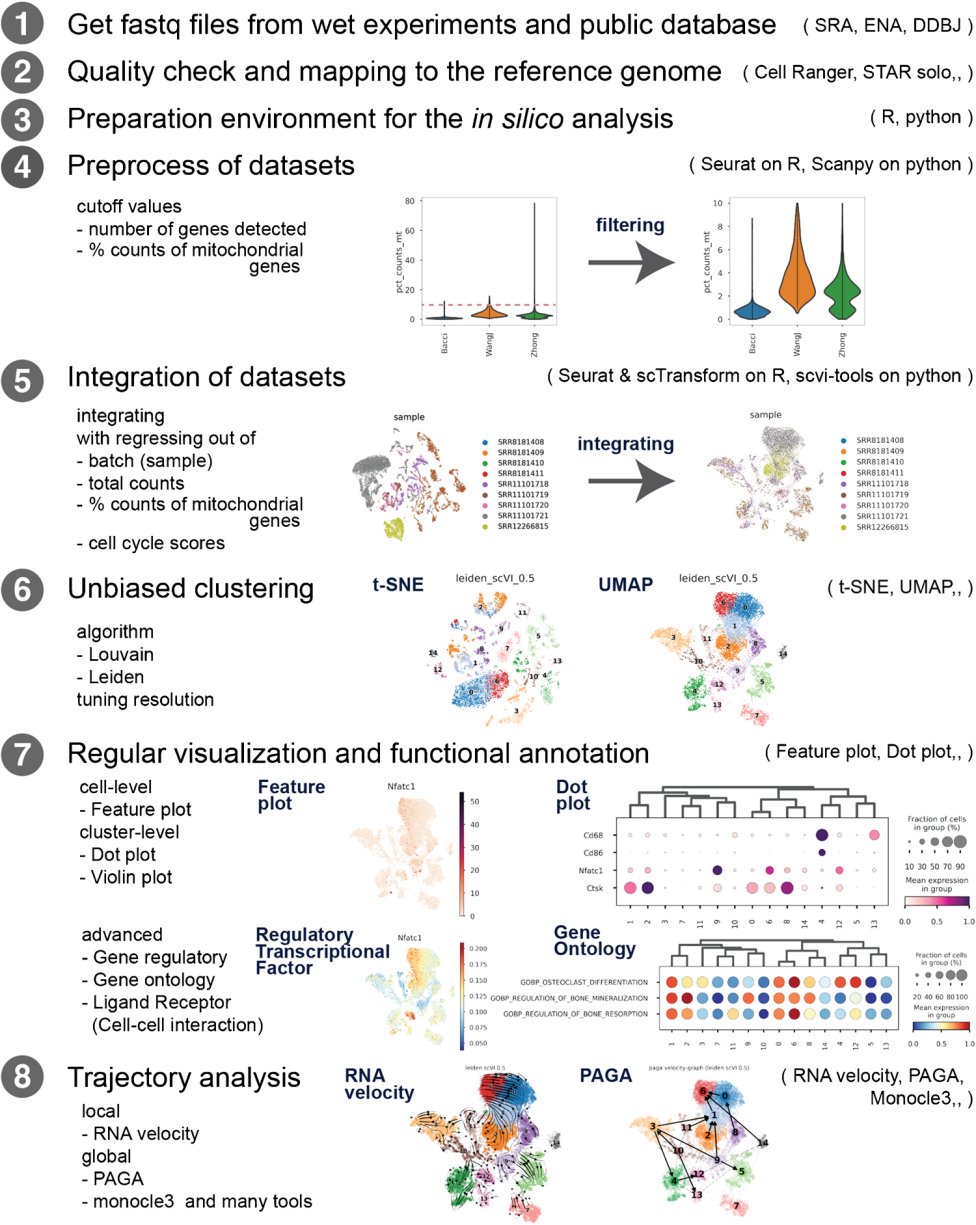
Standard workflow of in silico scRNA-seq analysis. All figures were made from publicly available datasets (SRR8181408, SRR8181409, SRR8181410, SRR8181411, SRR11101718, SRR11101719, SRR11101720, SRR11101721, and SRR12266815) [13–15]

The standard workflow of in silico scRNA-seq analysis is summarized in Fig. 1. The scRNA-seq packages and tools recommended by the authors are summarized in Table 1. These tools were selected primarily because of their usability and widespread use.

**Table 1:** List of packages or tools recommended by the authors

Package or tool	First author	Year	Language	Title or explanation	Ref
Step 1. Obtain FASTQ files from public database					
fasterq-dump	The SRA Toolkit Development Team	2023	C	extracting data in FASTQ- or FASTA-format from SRA-accessions	[16]
parallel-fastq-dump	Valieris	2021	Python	Speed up the process by dividing the work into multiple threads	[17]
Step 2. Quality check and mapping to the reference genome					
Cell Ranger	Zheng	2017		Massively parallel digital transcriptional profiling of single cells	[20]
STARsolo	Kaminow	2021	C	STARsolo: accurate, fast, and versatile mapping/quantification of single-cell and single-nucleus RNA-seq data	[21]
Step 3. Preparation environment for the in silico analysis					
R	R Core Team	2023		R: A Language and Environment for Statistical Computing	[22]
Tidyverse	Wickham	2019	R	Welcome to the Tidyverse	[23]
ggplot2	Wickham		R	Elegant Graphics for Data Analysis	[24]
Python3	Van Rossum	2009		Python 3 Reference Manual	[25]
Matplotlib	Hunter	2007	Python	Matplotlib: A 2D graphics environment	[26]
seaborn	Waskom	2021	Python	seaborn: statistical data visualization	[27]
Step 4. Preprocess of datasets					
Seurat 4	Hao	2021	R	Integrated analysis of multimodal single-cell data	[28]
Seurat 5	Hao	2022	R	Dictionary learning for integrative, multimodal, and scalable single-cell analysis	[29••]
sctransform	Hafemeister	2019	R	Normalization and variance stabilization of single-cell RNA-seq data using regularized negative binomial regression	[30]
Scanpy	Wolf	2018	Python	SCANPY: large-scale single-cell gene expression data analysis	[31]
scverse	Virshup	2023	Python	The scverse project provides a computational ecosystem for single-cell omics data analysis	[32]
Step 5. Dataset integration					
Seurat 5	Hao	2022	R		[29••]
scvi-tools	Gayoso	2022	Python	A Python library for probabilistic analysis of single-cell omics data	[34•]
benchmark study	Luecken	2022		Benchmarking atlas-level data integration in single-cell genomics	[33•]
Step 6. Unbiased clustering					
t-SNE	van der Maaten	2008		Visualizing Data using t-SNE	[35]
UMAP	Leland McInnes	2020		UMAP: Uniform Manifold Approximation and Projection for Dimension Reduction	[36]
Step 7. Functional annotation					
AUCell	Aibar	2016	R, Python	AUCell: Analysis of “gene set” activity in single-cell RNA-seq data	[38]
SCENIC	Aibar	2017	R	SCENIC: single-cell regulatory network inference and clustering	[39]
pySCENIC	Van de Sande	2020	Python	A scalable SCENIC workflow for single-cell gene regulatory network analysis	[39, 40]
decoupleR	Badia	2022	R, Python	decoupleR: ensemble of computational methods to infer biological activities from omics data	[41•]
CellAssign	Zhang	2019	Python	Probabilistic cell-type assignment of single-cell RNA-seq for tumor microenvironment profiling	[42]
Nitchenet	Browaeys	2020	R	NicheNet: modeling intercellular communication by linking ligands to target genes	[44]
Omnipath	Turei	2021	R, Python	Integrated intra- and intercellular signaling knowledge for multicellular omics analysis	[45]
scTensor	Tsuyuzaki	2019	R	Uncovering hypergraphs of cell–cell interaction from single cell RNA-sequencing data	[46]
Cellular interaction review	Armingol	2021		Deciphering cell–cell interactions and communication from gene expression	[43]
Step 8. Trajectory analysis					
Velocyto	La Manno	2018	Python, R	RNA velocity of single cells	[47]
scVelo	Bergen	2020	Python	Generalizing RNA velocity to transient cell states through dynamical modeling	[48•]
Dynamo	Qiu	2022	Python	Mapping transcriptomic vector fields of single cells	[49]
Monocle 3	Trapnell	2014	R	The dynamics and regulators of cell fate decisions are revealed by pseudotemporal ordering of single cells	[50]
PAGA	Wolf	2019	Python	graph abstraction reconciles clustering with trajectory inference through a topology preserving map of single cells	[51]
benchmark study	Saelens	2019		A comparison of single-cell trajectory inference methods	[52•]

### Step 1. Obtain FASTQ files from wet experiments and public database

There are two ways to obtain FASTQ files. One is to utilize public resources and the other is to perform wet experiments. The combinatorial approach has become more common with the increase in public datasets.

The first method for obtaining FASTQ files is to download them from a public database. sequence read archive (SRA), European nucleotide archive (ENA), and DDBJ sequence read archive (DRA) are the three major archives of sequencing datasets. The DDBJ search website is useful for finding the SRR number of datasets related to the project. The same project in one’s own experiments sometimes spares you from conducting expensive reproductive experiments. Public datasets were also used to increase the scale of scRNA-seq experiments. Integrative analyses are recommended for the following two reasons. First, a greater variety of cells often makes cell annotation easier. Second, integrative analysis with datasets from other research groups reduces biases related to the procedures of the group and increases the external validity of the experiments.

It takes considerable time and computer resources to download heavy FASTQ files of tens of gigabytes. fasterq-dump in the SRA toolkit [16], which is a fast version of fastq-dump, is commonly used to speed up downloading. parallel-fastq-dump [17] splits fastq files and downloads them by palletization of the process.

The second method is to perform a scRNA-seq experiment on one’s own. Although wet experiments are beyond the scope of this article, attention should be paid to the process of preparing cell suspensions. This is because the selection bias in wet experiments affects the results of in silico scRNA-seq. Less bias between samples makes the integration of scRNA-seq datasets easier.

Cell suspension preparation is the first step of droplet-based scRNA-seq. To smoothen the preparation step, we should repeatedly practice the entire preparation process and stabilize protocols.

When dissociating cells from solid tissue, a variety of healthy cells should be maintained and as many dead cells should be excluded as possible. Cell-sorting techniques using flow cytometry and magnetic devices are useful. After making a cell suspension, cell aggregation often occurs, which may cause problems in fluid-based sorting and should be loosened by pipetting.

After making cell suspensions, we performed highly elaborate library construction according to the manufacturer’s protocol like chromium (10 × genomics, Pleasanton, CA, U.S.). The number of living cells is important for the first chromium step. Instead of droplet-based sequencing, traditional plate-based sequencing after cell sorting, for example, SMART-seq® Single Cell Kit (Takara Bio, San Jose, CA, U.S.) [18], and RT-RamDA® cDNA Synthesis Kit (TOYOBO, Osaka, Japan) [19], are powerful tools for full-length total sequencing. After sequencing the constructed library, the FASTQ files are obtained.

### Step 2. Quality check and mapping to the reference genome

Cell Ranger is a useful pipeline to align outputted fastq files by chromium, on the prebuilt reference genome and make the folder of ready-to-use matrix files for the downstream analysis [20]. The Cell Ranger version and the reference genome used should be included in the manuscript to help reproduce the analysis. Cell Ranger consumes substantial computer memory; therefore, enough memory and storage more than the required level should be prepared. When using a public computer, the impact of load on the common space should be considered.

The authors preferred STARsolo [21], which is a single-cell version of the common aligner STAR. SMART-seq or Drop-seq datasets, other than chromium, can be processed using the same protocol. In addition, the same reference genome as that used in bulk RNA-seq can be used with simple arguments by STARsolo. Unlike Cell Ranger, the library construction protocol, including the chromium chemistry version, should be specified as a variable.

STARSolo offers three advantages. First, STARsolo can be adapted for other scRNA-seq datasets such as SMART-seq. Second, the mapping time is shorter than that of CellRanger. Third, this is the most important reason, the same reference genome as the usual bulk RNA-seq can be used.

When performing integrative analysis with other experiments of yours or public datasets by other groups, the same mapping protocol should be performed to avoid a mismatch of the reference genome. Repeat mapping on the reference genome is frequently performed. This is partially because Cell Ranger is frequently updated.

### Step 3. Preparation environment for the in silico analysis

Some vendors have provided browser-based analytical tools. These readily available tools are useful for checking quickly whether wet experiments are successful. However, it is too difficult to perform advanced analysis, including cell interaction, and to produce images with publication quality using only these tools. This is why researchers and reviewers should be familiar with in silico analysis.

R language [22] is commonly used in statistical science and bioinformatics analyses. Tidyverse project [23] provides several powerful toolkits for handling datasets with simple syntax. In particular, ggplot2 [24] increases the visibility of graphs and ensures reproducibility, which is important in science.

Updating R and the packages sometimes yields different UMAP or clustering results. Major R updates require package reinstallation. Although we do not want to update these versions, version conflicts between packages do not allow us to change only problematic packages, but enforce updating all packages. The results without big picture changes with different versions, in which minor detailed changes are allowed, should be stated in the manuscript.

Memory usage should be cared for when using R. Regardless of the PC setup, the memory consumption of R can cause sudden crashes. This is a good practice for saving files and images. It is also important to delete the unused variables and intermediate files.

Taking fashionable machine learning methods into the study, converting platform R to Python3 is considered because of the large memory requirement [25]. The main feature of Python is its numerous modules, including deep learning. Another feature is the creation of a virtual environment with modules required for each project to avoid version conflicts between the packages. Matplotlib [26] and Seaborn [27] support data visualization. Based on the author’s experience, switching from R to Python requires practice.

### Step 4. Preprocess of datasets

Seurat [28] is a core package for processing and normalizing scRNA-seq datasets. Seurat has been developed to integrate multiple datasets. In the latest version 5 [29••], we can choose an integration method including sctransform [30]. In Python, Scanpy [31] in scverse project [32] is the core package for processing the datasets. It is not difficult to handle fundamental packages in both R and Python because tutorials are available online and many virtual workshops are available. Core preprocessing: Filtering and normalization are almost the same regardless of the package.

The total number of detected genes, percentage of counts on mitochondrial genes, and sometimes ribosomal genes are common indicators for cutting off dead cells or poorly sequenced cells. These cutoff values should be listed in the manuscript. Different technologies for library construction typically result in different levels of these indicators. Different levels are often observed, even with the same technology. The same cutoff value is recommended for making posterior calculation easy; however, different cutoff values may be accepted for each dataset.

The selected cells are then normalized to compare RNA expression between cells. Additional normalization of the total number of reads should be performed because of the low sensitivity of single-cell sequencing from a low amount of RNA. Percentages of mitochondrial gene count and cell cycle scores are usually regressed out for normalization. Although cell cycle scoring and assignment of the cell cycle state for each cell are performed routinely by Seurat, cell cycles are predicted by comparing the mRNA expression of cell cycle genes. When analyzing datasets in which the cell cycle is highly activated, cell cycle regression may be unnecessary.

### Step 5. Dataset integration

It is common to handle multiple datasets; however, it is difficult to integrate datasets with different expression levels. Various integration methods have been devised and are currently under development. The latest method should be considered at the time of submission because the choice of method leads to different results. Benchmark studies on integration are useful for selecting packages [33•]. The latest version of Seurat allows the selection of various integration methods [29••]. Scverse projects provide scvi-tools to perform probabilistic analysis, particularly when integrating datasets [34•]. Whether the results of the integration are correct should be examined from the perspective of wet scientists.

### Step 6. Unbiased clustering

To understand the heterogeneous gene expression patterns at a glance, dimension reduction with tSNE [35] and UMAP [36] is performed. The distribution of datasets, mitochondrial percentage, and cell cycle are indicators of the successful integration of the datasets. Unbiased clustering after dimension reduction makes it possible to depict the cell populations. The resolution must be tuned by the authors to adapt the assumed cell types, although some tools for the automatic determination of cluster numbers have been proposed [37].

Maps derived from scRNA-seq datasets are built based on RNA expression patterns and do not always fit the standard biological view. Not only local structures but also the whole picture often change according to the method. For example, small islands and their connectivity to a main island can be easily transformed. The robustness of in silico results should be examined, especially when discussing minor cell populations. Insufficient batch-effect elimination often yields distinct clusters. In some cases, more cells are required to reach a conclusion. Public datasets are used as external references to reduce researcher bias.

### Step 7. Regular visualization and functional annotation

In scRNA-seq, gene expression and cellular functions are explained at two levels: cell and cluster or group. Feature plots explain gene expression cell by cell on the same map. Continuous changes in the levels are easily depicted. Dot plots and violin plots are used to summarize the expression levels group by group. Dot plots are recommended for showing sets of genes within a limited space.

Cell types and their functions are determined by considering sets of gene expression, sometimes called gene set activities. AUCell is useful for calculating the gene set activity score [38], and with the same algorithm, we can predict upstream transcription factor activity using SCENIC or pySCENIC [39, 40]. decoupleR [41•] enables the use of multiple ensemble annotative methods including AUCell. Automatic cell annotation using the deep learning method is implemented using CellAssign [42].

Intercellular interactions can be predicted using paired gene expression in different groups of cells, including ligand receptor (LR) interactions. Several types of tools have been proposed [43]. Nitchenet [44] considers downstream pathways including receptors to transcriptional factors. The LR pair database directly affects the results of the LR analysis. Omnipath project provides large well-organized references [45]. With abundant computer memory, tensor-based cell communication analysis between more than two cell types provides further LR relationships [46].

### Step 8. Trajectory analysis

RNA velocity is a well-known concept for predicting local changes in the cellular state from the spliced/unspliced ratio of sequenced reads and is implemented as Velocyto [47]. Streamline visualization can be illustrated by a generalized version of Velocyto called scVelo [48•]. Plots with arrows are attractive; however, RNA velocity tools are still under development [49]. Trajectory analysis provides just supportive evidence for cellular pathways.

Monocle2 or monocle3 in R [50], and PAGA in Python [51] are commonly used to draw global trajectory lines based on gene expression. To choose the appropriate tool for each analysis, rough topological characteristics, such as cycle, linear, branch, tree, and disconnection, should be presumed before trajectory analysis. Dynguidelines project provides a clue for selecting appropriate trajectory tools for each analysis [52•].

## Conclusion

scRNA-seq provides insight into the diversity of cell populations. However, the preprocessing and integrative steps for multiple datasets remain controversial. The honest manifestation of the fundamental steps makes the study reproducible.

Reviewers of scRNA-seq research should first check these basic points (summarized in Table 2) and then discuss whether the biological interpretation is reasonable. There have been scRNA-seq studies with insufficient replicates. However, in the era when there are plenty of public scRNA-seq fastq files, investigators’ procedures should be examined for propriety and external validity.

**Table 2:** Key consideration when reviewing a scRNA-seq data analysis

Usage of published tools at the time of submission (not the time of review)
The number of cells was sequenced and analyzed in each experiment
Sufficient replication to support the claim
Cutoff values to eliminate dead or badly sequenced cells
Dataset integration method mainly considering the batch effect of each sample
Unbiased clustering examined by biological interpretation
Optional (Not mandatory) trajectory analyses
Cell type annotation referring to marker genes and gene ontology term

The use of scRNA-seq analysis has continued to evolve rapidly. Discussion between investigators and reviewers should be performed within the scope of the methods at the time of submission. Better use of this innovative technique will enhance our biological knowledge.
