# Correction: Practical Compass of Single-Cell RNA-Seq Analysis

**DOI:** 10.1007/s11914-024-00861-7

**Published:** 2024-02-19

**Authors:** Hiroyuki Okada, Ung-il Chung, Hironori Hojo

**Affiliations:** 1 Center for Disease Biology and Integrative Medicine, Graduate School of Medicine, The University of Tokyo, Bunkyo-Ku, Tokyo, 113-8655 Japan; 2 Department of Orthopaedic Surgery, The University of Tokyo, Tokyo, Japan; 3 Department of Oral Medicine, Infection, and Immunity, Harvard School of Dental Medicine, Boston, MA 02115 USA; 4 Department of Bioengineering, Graduate School of Engineering, The University of Tokyo, Tokyo, Japan

**Correction: Current Osteoporosis Reports**

10.1007/s11914-023-00840-4

In Table 1 of this article, each ref. number is off by one less than the correct number.

The original article has been corrected.

